# Notch signalling in the paraxial mesoderm is most sensitive to reduced *Pofut1 *levels during early mouse development

**DOI:** 10.1186/1471-213X-9-6

**Published:** 2009-01-22

**Authors:** Karin Schuster-Gossler, Belinda Harris, Kenneth R Johnson, Jürgen Serth, Achim Gossler

**Affiliations:** 1 Institute for Molecular Biology, Medizinische Hochschule Hannover, Carl-Neuberg-Str. 1, D-30625, Germany; 2 The Jackson Laboratory, 600 Main Street, Bar Harbor, Maine, USA; 3 Clinic for Urology, Medizinische Hochschule Hannover, Carl-Neuberg-Str. 1, D-30625, Germany

## Abstract

**Background:**

The evolutionarily conserved Notch signalling pathway regulates multiple developmental processes in a wide variety of organisms. One critical posttranslational modification of Notch for its function in vivo is the addition of O-linked fucose residues by protein O-fucosyltransferase 1 (POFUT1). In addition, POFUT1 acts as a chaperone and is required for Notch trafficking. Mouse embryos lacking POFUT1 function die with a phenotype indicative of global inactivation of Notch signalling. O-linked fucose residues on Notch can serve as substrates for further sugar modification by Fringe (FNG) proteins. Notch modification by Fringe differently affects the ability of ligands to activate Notch receptors in a context-dependent manner indicating a complex modulation of Notch activity by differential glycosylation. Whether the context-dependent effects of Notch receptor glycosylation by FNG reflect different requirements of distinct developmental processes for O-fucosylation by POFUT1 is unclear.

**Results:**

We have identified and characterized a spontaneous mutation in the mouse *Pofut1 *gene, referred to as "compact axial skeleton" (*cax*). *Cax *carries an insertion of an intracisternal A particle retrotransposon into the fourth intron of the *Pofut1 *gene and represents a hypomorphic *Pofut1 *allele that reduces transcription and leads to reduced Notch signalling. *Cax *mutant embryos have somites of variable size, showed partly abnormal *Lfng *expression and, consistently defective anterior-posterior somite patterning and axial skeleton development but had virtually no defects in several other Notch-regulated early developmental processes outside the paraxial mesoderm that we analyzed.

**Conclusion:**

Notch-dependent processes apparently differ with respect to their requirement for levels of POFUT1. Normal *Lfng *expression and anterior-posterior somite patterning is highly sensitive to reduced POFUT1 levels in early mammalian embryos, whereas other early Notch-dependent processes such as establishment of left-right asymmetry or neurogenesis are not. Thus, it appears that in the presomitic mesoderm (PSM) Notch signalling is particularly sensitive to POFUT1 levels. Reduced POFUT1 levels might affect Notch trafficking or overall O-fucosylation. Alternatively, reduced O-fucosylation might preferentially affect sites that are substrates for LFNG and thus important for somite formation and patterning.

## Background

The evolutionarily conserved Notch signalling pathway mediates direct cell-to-cell communication in a wide variety of developmental contexts in different species and regulates cell-fate decisions, proliferation and apoptosis [1-6]. Notch genes encode large transmembrane proteins that act at the surface of a cell as receptors for proteins encoded by the Delta and Serrate (Jagged in mammals) genes. Like Notch, the transmembrane ligands Delta and Serrate have a variable number of EGF-like repeats in their extracellular domains [7-9]. Upon ligand binding, the intracellular portion of Notch is proteolytically released, translocates to the nucleus, and by complexing with a transcriptional regulator of the CSL family, RBP-jκ in mouse, activates transcription of target genes [10-16].

Notch modification by O-linked fucose residues is essential for Notch signalling in vivo both in *Drosophila *and mammals [17-19]. O-linked fucose residues are attached to specific Ser or Thr residues in epidermal growth factor-like sequence repeats of Notch [20,21]. The transfer of fucose to these residues is catalyzed by protein O-fucosyltransferase 1 (POFUT1), which is encoded by Ofut1 in *Drosophila *and *Pofut1 *in mammals [22]. In addition, in certain cell types POFUT1 acts independently from its fucosyltransferase activity as a chaperone and is required for Notch folding and presentation on the cell surface [23,24]. *Pofut1 *null (*Pofut1*^*tm*1*Pst*/*tm*1*Pst*^) mutant mouse embryos are severely growth retarded on E9.5 and die around E10 with a phenotype resembling embryos lacking the common downstream effector RBP-jκ or presenilins, which are required for the release of the intracellular domains of Notch receptors, suggesting that Notch signalling is globally inactivated through all four mammalian Notch receptors [19].

O-linked fucose residues on EGF repeats serve as substrates for further modification by Fringe (FNG) proteins, fucose-specific beta1, 3 N-acetylglucosaminyltransferases that modify Notch in the trans-Golgi and modulate the interactions of Notch receptors with their ligands [20,21]. Notch modification by Fringe differentially affects the ability of ligands to activate Notch receptors in a context-dependent manner [25-27]. For example, in the *Drosophila *wing disc Fringe potentiates a cell's ability to respond to Delta and inhibits its ability to respond to Serrate [27], whereas in the presomitic mesoderm of mouse embryos Lunatic fringe (LFNG) appears to attenuate Delta1-like (DLL1)-mediated activation of Notch1 [26]. In vitro LFNG may enhance DLL1-mediated signalling and inhibits Jagged1-mediated signalling through Notch1, or potentiate both Jagged1- and DLL1-mediated signalling via Notch2 [25] indicating a complex modulation of Notch activity by differential glycosylation.

The context-dependent effects of Notch receptor glycosylation by LFNG suggest that different developmental processes might have different requirements for O-fucosylation by POFUT1. However, this cannot be assessed due to the early embryonic lethality of *Pofut1 *null mutants. Here, we identify a spontaneous mutation in the *Pofut1 *gene that leads to a hypomorphic allele. Mice homozygous for this allele display defects in the axial skeleton consistent with the known patterning functions of Notch in somitogenesis, but have no apparent defects in other early Notch-dependent processes such as left-right determination, vascular remodelling or neuronal differentiation. Our results suggest that aspects of somite formation and patterning that depend on Notch function are processes that are most sensitive to the level of POFUT1 in early mammalian embryos.

## Results

### *Cax*, a novel spontaneous mouse mutation affecting axial skeleton development

**Figure 1:**
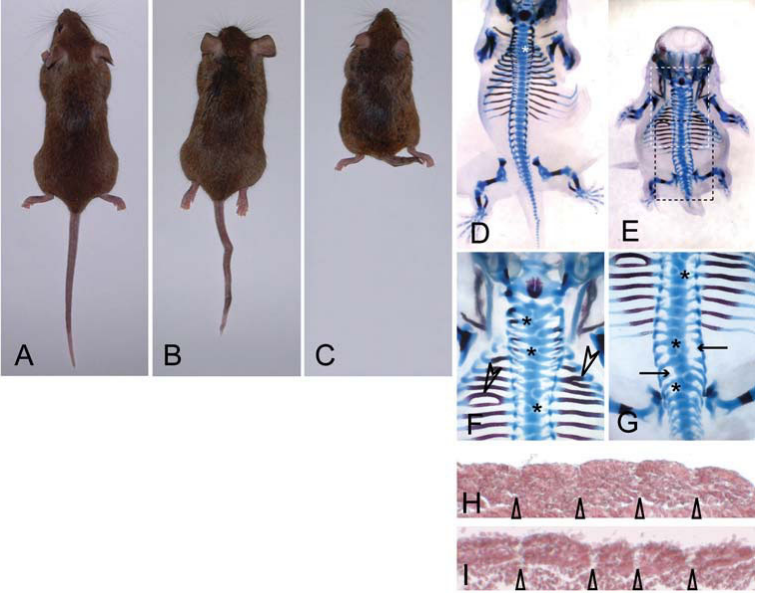
**External phenotype and skeletal and somite defects in *cax *mutant mice**. (A-C) Examples of homozygous mutants demonstrating the variable phenotype in the backcross progeny. (D-G) Skeletal preparations of E15.5 embryos showing that even externally apparently normal mice have skeletal defects (D), and demonstrating various defects such as split vertebrae (asterisks in D, F, G), rib fusions and bifurcations (arrowheads in F), reduced or missing pedicles (arrows in G), and axial truncations (E). White and black boxes in (E) indicate the regions shown enlarged in (F) and (G), respectively. (H, I) Sections of E9.5 *cax *mutant embryos showing distinct somite borders (indicated by arrowheads) and somites of variable size.

A new recessive autosomal mutation named "compact axial skeleton" (*cax*), causing kinky and shortened tails and shortened body length, arose spontaneously in a breeding colony of C3H/HeJ mice at The Jackson Laboratory. On the C3H/HeJ background the *cax *mutation causes severe shortening of the tail and body axis, whereas on the mixed genetic background of our linkage cross the phenotype was variable, and ranged from normal external appearance to almost complete absence of the tail and shortened body axis (Figure 1A–C). Most likely the variability seen in the backcross progeny is attributable to strain background effects. Skeletal preparations showed that even externally normal mice carried mild malformations of the vertebral column (Figure 1D, and data not shown). Skeletal malformations were found along the entire length of the vertebral column (Figure 1E) and included fused ribs (arrowheads in Figure 1F), reduced or missing pedicles (arrows in Figure 1G) and split or hemi-vertebrae (asterisks in Figure 1D, F, G). At earlier stages, mutant embryos had clearly discernable somite borders, however, in some embryos somite size varied considerably (Figure 1H, I, and data not shown). The observed defects are indicative of defective somite formation and compartmentalization suggesting that the *cax *mutation affects a gene involved in early somite patterning.

### Identification of *Pofut1 *as the gene affected by the *cax *mutation

**Figure 2:**
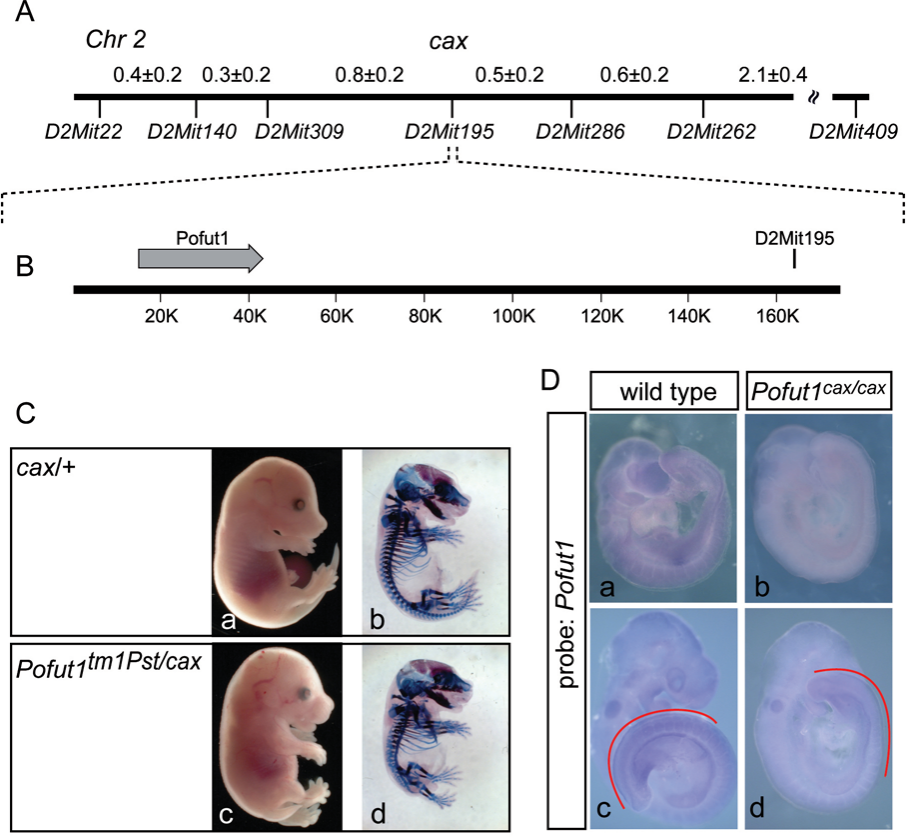
**The *cax *mutation is an allele of *Pofut1***. (A) Genetic map position of chromosome 2 around the *cax *mutation based on analysis of 1339 backcross progeny. Markers and genetic distances are indicated below and above the map, respectively. (B) Physical map of the *Pofut1 *genomic region and location of *D2Mit195*. (C) Normal external (a) and skeletal (b) phenotype of E15.5 heterozygous *cax*/+ mice, and shortened body axis (c) and defective axial skeleton (d) in *Pofut1*^*tm*1*Pst*/*cax *^double heterozygous mice. (D) Reduced levels of *Pofut1 *mRNA in *Pofut1*^*cax*/*cax *^mutants (b, d) compared with wild type embryos (a, c) on E9 (a, b) and E9.5 (c, d) detected by in situ hybridization under identical hybridization and staining conditions. Red lines in c and d indicate the PSM and somites.

To identify the gene affected by the *cax *mutation we mapped *cax *genetically by linkage analysis of the mutant phenotype with segregating chromosome markers. First, *cax *was assigned to chromosome 2 in an interval between the microsatellite markers *D2Mit22 *and *D2Mit409 *by analysis of 62 mutant F2 offspring from an intercross of (C3H/HeJ-*cax/cax *× CAST/Ei+/+) F_1 _hybrids. A fine genetic map was established by analysis of 1339 N2 mutant progeny from a backcross of F1 hybrids to *cax/cax *mutants. The locus order and inter-locus distances (in cM ± SD) for the candidate gene region deduced from our analysis is *D2Mit22*-(0.4 ± 0.2)-*D2Mit140*-(0.3 ± 0.2)-*D2Mit309*-(0.8 ± 0.2)-[*D2Mit195, cax*]-(0.5 ± 0.2)-*D2Mit286*-(0.6 ± 0.2)-*D2Mit262*-(2.1 ± 0.4)-*D2Mit409 *(Figure 2A). Analysis of the mouse genome sequence showed that *D2Mit195 *(Chr 2 position 153.082 Mb, NCBI build 36), which did not recombine with *cax*, resides approximately 120 kb downstream of the *Pofut1 *gene (Chr 2 position 152.933–152.962 Mb; Figure 2B). *Pofut1 *appeared as an appealing candidate since O-fucose modification of Notch is essential for receptor function [18,19], and Notch signalling is required for normal somite formation and patterning [28]. To directly test whether *cax *affects *Pofut1 *we crossed homozygous *cax *mutants with heterozygous mice carrying a targeted null allele of *Pofut1*, *Pofut1*^*tm*1*Pst*^[19]. Mice carrying one copy of the *cax *mutation and one *Pofut1*^*tm*1*Pst *^allele had a significantly shortened body axis and axial skeleton defects (Figure 2C c, d), indicating that the recessive *Pofut1 *null allele does not complement the *cax *mutation and identifying *cax *as a novel allele of *Pofut1*, which we refer to as *Pofut1*^*cax *^from hereon.

To address whether the *Pofut1*^*cax *^mutation affects the *Pofut1 *coding sequence, we amplified *Pofut1 *cDNA from mRNA purified from homozygous *Pofut1*^*cax *^kidney and C3H wild type mice, and sequenced at least two independently generated cDNA clones. No mutation in the coding sequence was detected (data not shown), suggesting that *Pofut1*^*cax *^affects *Pofut1 *transcription. Consistent with this idea, a probe from the coding region revealed by *in situ *hybridization overall reduced levels of *Pofut1 *transcripts in a considerable portion of homozygous *Pofut1*^*cax *^mutant embryos (Figure 2D, b and 2d, and data not shown) that are also apparent in the PSM and developing somites (compare regions indicated by red lines Figure 2D, c and 2d).

### Identification of the *Pofut1*^*cax *^mutation

**Figure 3:**
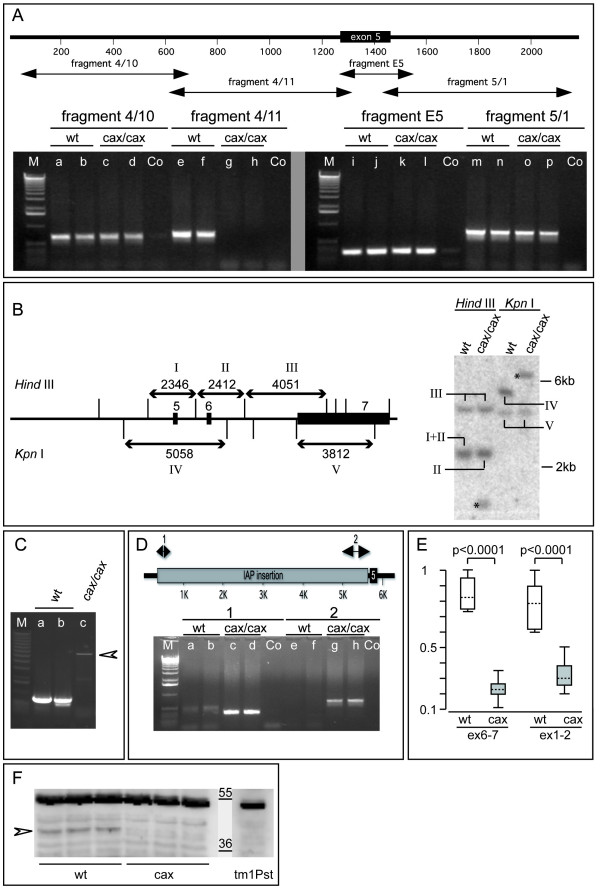
***Pofut1*^*cax *^contains an IAP insertion**. (A) Map of the region flanking exon5 and PCR-amplified DNA fragments. Mutant DNAs did not produce fragment 4/11 (g, h) but all others (c, d, k, l, o, p) as wild type (wt) (a, b, e, f, i, j, m, n). (B) Map of the 3' region of *Pofut1*, and Southern blot of wt and mutant DNA hybridized with a cDNA probe containing exon5, 6, and a 5' portion of exon7. Restriction sites and fragments (arrows, labelled by Roman numerals in the scheme and blot) are indicated above and below the map. Asterisks indicate mutant-specific fragments. (C) Long range PCR amplifying fragment 4/11 gave a mutant product (c) larger than wt (a, b). (D) Insertion site map, and junction fragments (1 and 2) amplified with mutant (c, d, g, h) but not wt (a, b, e, f) DNA. Co: water, M: 1 kb ladder. (E) Relative *Pofut1 *mRNA levels in E10.5 wt (n = 6; white boxes) and mutant embryos (n = 12; gray boxes) determined by exon 6–7 and exon 1–2 amplification. Boxed areas contain 50% of all values. Stippled lines indicate Median expression, whiskers of the boxed areas the total range of values. (F) POFUT1 protein (arrowhead) detected in E9.5 wt embryo extracts was reduced in *cax *mutants, and absent from *Pofut1*^*tm*1*Pst*/*tm*1*Pst*^. Due to the lower amount of protein in a single growth retarded *Pofut1*^*tm*1*Pst*/*tm*1*Pst *^embryo this lane shows a longer exposure using the non-specific 55 kDa band as an adjustment. Bars indicate molecular weight markers (kDa).

To identify potential structural alterations at the *Pofut1 *locus that might cause the mutant phenotype we scanned 20 kb upstream of the ATG as well as the whole intragenic region of the *Pofut1*^*cax *^allele by PCR. Primers were designed to amplify overlapping DNA fragments between approximately 500 and 1000 bp in length, and genomic DNAs prepared from two wild type C3H and two mutant *Pofut1*^*cax*/*cax *^mice were used as templates. With the exception of one primer pair that consistently failed to amplify a fragment of the 3' end of intron 4 from mutant DNA (fragment 4/11 in Figure 3A) all PCR reactions amplified fragments of indistinguishable size from wild type and mutant DNA. This suggested that the *Pofut1*^*cax *^mutation disrupts the integrity of the *Pofut1 *locus in this region. Consistent with this idea, Southern blot analyses using a cDNA probe that contained exons 5 and 6, and the 5' portion of exon 7 revealed restriction fragment length polymorphisms between wild type and mutant genomic DNA (Figure 3B, and data not shown).

The primers used to amplify fragment 4/11 are located in fragments 4/10 and fragment E5, and should therefore be present in mutant DNA (Figure 3A, lanes c, d, and k, l). Thus, we reasoned that an insertion into fragment 4/11 might prevent amplification of this fragment from mutant DNA by conventional PCR. Therefore, we employed long range PCR with fragment 4/11 primers, and amplified an approximately 6 kb fragment from mutant DNA (arrowhead in Figure 3C). Sequencing of this fragment identified an intracisternal A particle (IAP) insertion into *Pofut1*^*cax *^intron 4 (Figure 3D). The presence of the insertion in the *Pofut1*^*cax *^genomic DNA was independently verified by PCR reactions that specifically amplify the junction fragments (lanes c, d and g, h in Figure 3D). In E10.5 *cax *mutant embryos (n = 12) *Pofut1 *mRNA levels were reduced to approximately 25% of wild type (n = 6) levels (Figure 3E) as determined by TaqMan real time PCR. Western blot analyses with anti-POFUT1 antibodies [29] showed also reduced POFUT1 protein levels in cell lysates from *Pofut1*^*cax *^embryos (Figure 3F). The protein reduction could not precisely be determined due to the normally low levels of endogenous POFUT1 in embryos and additional background bands. Thus, the *Pofut1*^*cax *^allele carries an insertion of a retrotransposon that most likely underlies variably reduced *Pofut1 *mRNA and POFUT1 protein levels. This variable reduction might also explain the variable phenotype of *cax *mutant embryos and mice.

### Effects of the *Pofut1*^*cax *^mutation on Notch dependent processes

**Figure 4:**
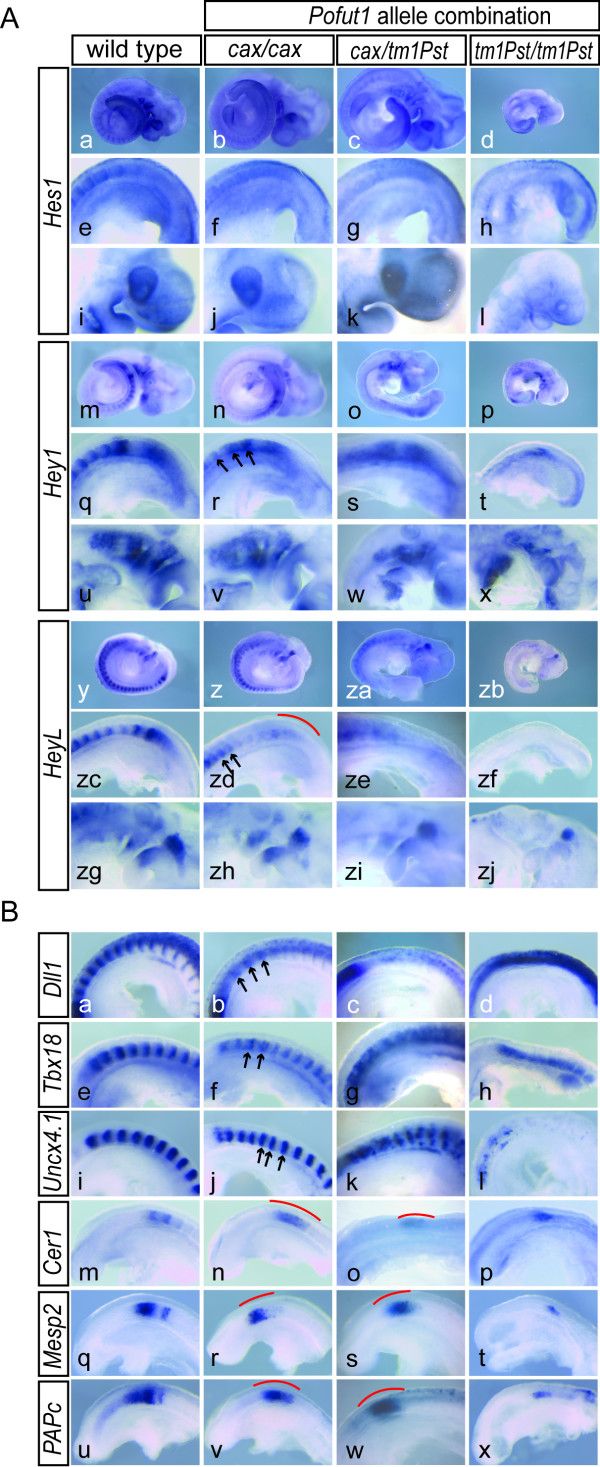
**Disturbed A-P somite polarity in *Pofut1*^*cax*/*cax *^embryos**. (A) WISH of E9.5 embryos with Notch targets. *Hes1*, *Hey1 *and *HeyL *expression is abnormal in the paraxial mesoderm of *Pofut1*^*cax*/*cax *^embryos (b, f; n, r; z, zd) compared to wild type (a, e; m, q; y, zc), largely normal in other regions (compare j, v, zh with i, u, zg), and overall severely reduced in *Pofut1*^*tm*1*Pst*/*tm*1*Pst *^embryos (d, h, l; p, t, x; zb, zf, zj). In *Pofut1*^*cax*/*tm*1*Pst *^embryos expression in the paraxial mesoderm is more severely disrupted (c, g; o, s; za, ze), *Hey1 *and *HeyL *expression in the branchial bars reduced (w, zi) and *Hes1 *expression in the optic vesicle apparently unaffected (k). (B) WISH on E9.5 embryos detecting A-P somite polarity. *Dll1*, *Tbx18 *and *Uncx4.1 *show abnormal expression in *Pofut1*^*cax*/*cax *^(b, f, j) and *Pofut1*^*cax*/*tm*1*Pst *^(c, g, k) somites compared to wild type (a, e, i). Stripes are weaker (arrows in b), irregularly spaced (arrows in f and j) or blurred with stronger abnormalities in heteroallelic embryos (c, g, k). *Pofut1*^*tm*1*Pst*/*tm*1*Pst *^embryos show increased expression of *Dll1 *(d) or *Uncx4*.*1 *(l) in the neural tube and no segment polarity (h, l). Instead of distinct stripes detected in wild type (m, q, u) *Pofut1*^*cax*/*cax *^and *Pofut1*^*cax*/*tm*1*Pst *^mutants exhibit blurry *Cer1*, *Mesp2*, and *Papc *expression (red lines in n, r, v, o, s, w). *Pofut1*^*tm*1*Pst*/*tm*1*Pst *^embryos show weaker and fuzzy expression (p, t, x). Red lines indicate regions of fuzzy gene expression, arrows point to stripes of abnormal expression.

To analyze in more detail how the *cax *allele affects Notch activity and Notch-dependent processes we first analyzed expression of the Notch target genes *Hes1*, *Hey1*, and *HeyL*, which are severely down-regulated in *Pofut1*^*tm*1*Pst*/*tm*1*Pst*^embryos (n = 3, respectively; Figure 4A d, h, l, p, t, x, zb, zf, zj). In *cax *mutant embryos expression of *Hes1*, *Hey1*, and *HeyL *was also obviously affected in the paraxial mesoderm and somites (Figure 4A f, r, zd). In contrast, expression in other domains, for example of *Hes1 *in the optic vesicle (compare Figure 4A i and 4j), and of *Hey1 *and *HeyL *in the branchial bars (compare Figure 4A u and 4v, and 4A zg and 4zh, respectively), was similar to wild type both with respect to expression levels and patterns. In the case of *Hes1*, the clearly visible stripes of expression in posterior somite compartments in wild type embryos (Figure 4A e) were not detected in *cax *mutants (n = 10; Figure 4A f)). Similarly, the strong distinct expression domains of *Hey1 *in posterior somite halves of wild type embryos (Figure 4A q) appeared fuzzy in *cax *mutants (n = 28; regions indicated by arrows in Figure 4A r), and expression in the PSM was reduced (Figure 4A r). In mutant embryos (n = 10) *HeyL *expression was reduced in the anterior PSM (red line in Figure 4A zd) and in newly formed somites, and expressed in a smaller expression domain in anterior somites (regions indicated by arrows in Figure 4A zd). The expression patterns of *Hes1*, *Hey1*, and *HeyL *in the paraxial mesoderm of embryos heteroallelic for the targeted null and the *cax *allele of *Pofut1 *were more severely disrupted (n = 3, respectively; Figure 4A g, s, ze), consistent with expected further reduced *Pofut1 *levels. In these embryos *Hes1 *expression in the optic vesicle (Figure 4A k) was not obviously affected, whereas *Hey1 *(Figure 4A w) and *HeyL *(Figure 4A zi) expression in the branchial bars appeared reduced, indicating that further reduction of *Pofut1 *levels in *Pofut1*^*cax*/*tm*1*Pst *^embryos affects Notch activity also outside the paraxial mesoderm.

Consistent with obvious alterations of *Hes1*, *Hey1*, and *HeyL *expression in the paraxial mesoderm also the expression patterns of genes that are important or indicative for somite patterning and polarity, and whose normal expression patterns depend on Notch activity, were altered in *Pofut1 *mutants. *Dll1 *expression was virtually abolished in the somites but upregulated in the neural tube of *Pofut1 *null mutant embryos (n = 3; Figure 4B d). Similarly, *Uncx4.1*, which is normally expressed in a regular pattern delineating posterior somite halves (Figure 4B i), was severely downregulated in somites of *Pofut1 *null mutants (n = 5; Figure 4B l), and expression of *Tbx18*, which normally delineates anterior somite halves (Figure 4B e), was expanded throughout somites (n = 3; Figure 4B h) indicating loss of somite polarity.

In *cax *mutants anterior-posterior somite patterning was also abnormal, as indicated by the reduced and broadened *Dll1 *expression domains in somites (n = 11; arrows in Figure 4B b), fuzzy expression domains and disorganized stripes of expression of *Tbx18 *(n = 27) and *Uncx4.1 *(n = 10; arrows in Figure 4B f, j). In heteroallelic *Pofut1*^*cax*/*tm*1*Pst *^embryos anterior-posterior somite patterning was more severely affected than in *Pofut1*^*cax*/*cax*^embryos: the somitic *Dll1 *stripes were essentially lost (Figure 4B c), *Tbx18 *expression domains were fuzzy and expanded (Figure 4B g) and, *Uncx4*.1 stripes were irregular and scrambled (Figure 4B k). Similarly, expression of *Cer1*, *Mesp2*, and *Papc *was disrupted by the *cax *mutation further supporting the notion that somite compartmentalization is affected. *Cer1*, whose expression is normally restricted to the anterior somite compartments of the prospective and most recently formed two somites (Figure 4B m), was expressed in one broad domain (n = 25; red line in Figure 4B n) similar to *Pofut1 *null mutants (n = 2; Figure 4B p). *Mesp2 *which is normally expressed in one or two distinct stripes of variable width (Figure 4B q, and data not shown) showed always only one not clearly delineated expression domain in *cax *mutant embryos (n = 31; red line in Figure 4B r), and the distinct domains of *Papc *expression (Figure 4B u) appeared as one blurred domain in *cax *(n = 36; red line in Figure 4B v). In heteroallelic *Pofut1*^*cax*/*tm*1*Pst *^embryos (n = 3, respectively), the expression patterns of these genes were similarly disrupted (Figure 4B o, s, w). In *Pofut1 *null mutants (n = 6) expression of these genes was severely downregulated in addition to their abnormal patterns (Figure 4B t, x).

**Figure 5:**
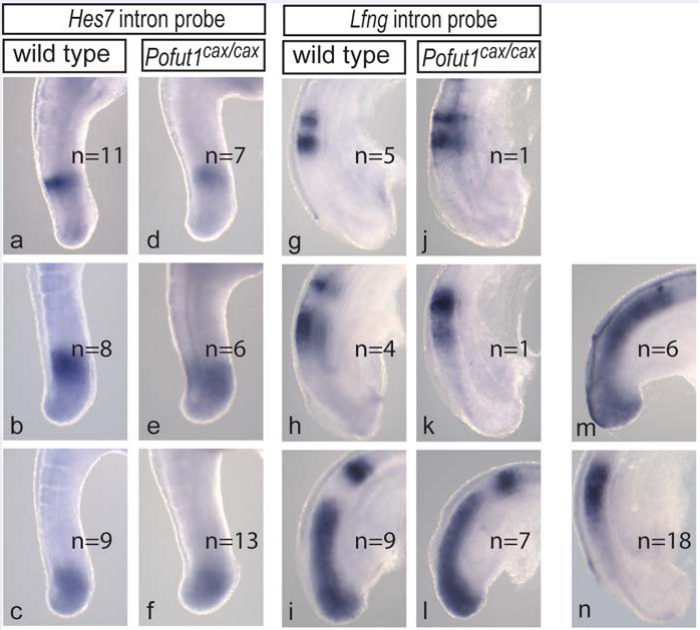
**Expression of cyclic Notch targets in the PSM**. *Hes7 *expression in *Pofut1*^*cax*/*cax *^mutant E10.5 embryos (d-f) was found in patterns similar to wild type embryos (a-c). In contrast, only 9/33 E9.5 embryos showed *Lfng *expression patterns (j-l) that could clearly be assigned to the distinct phases of expression seen in wild type embryos (g-i). The remainder showed either expression throughout the psm (m) or in one broad stripe anteriorly (n).

Since the establishment of somite polarity depends on the function of cyclic Notch pathway genes in the PSM [30-33] we analyzed the expression of *Hes7 *and *Lfng *in cax mutant embryos. *Hes7 *could be assigned to the three described phases both in wild type and *cax *mutant (n = 26) embryos albeit expression in cax mutants was only detected after prolonged staining suggesting that levels are reduced (Figure 5). Likewise, *Lfng *expression patterns reflecting the different phases were found in *cax *mutants (n = 9; Figure 5). In contrast, in additional 24 embryos we observed rather broad domains of expression in the PSM (Figure 5m, n) that could not be assigned to certain phases, suggesting that reduced POFUT1 levels in *cax *mutant embryos impinge on the dynamic expression of cyclic Notch target genes in particular on *Lfng*. Collectively, these data suggest that the reduced *Pofut1 *mRNA levels in *cax *mutant embryos attenuate Notch activity below a threshold required for patterning the presomitic mesoderm, leading to defects in regular somite spacing and compartmentalization that underlie the observed skeletal malformations.

**Figure 6:**
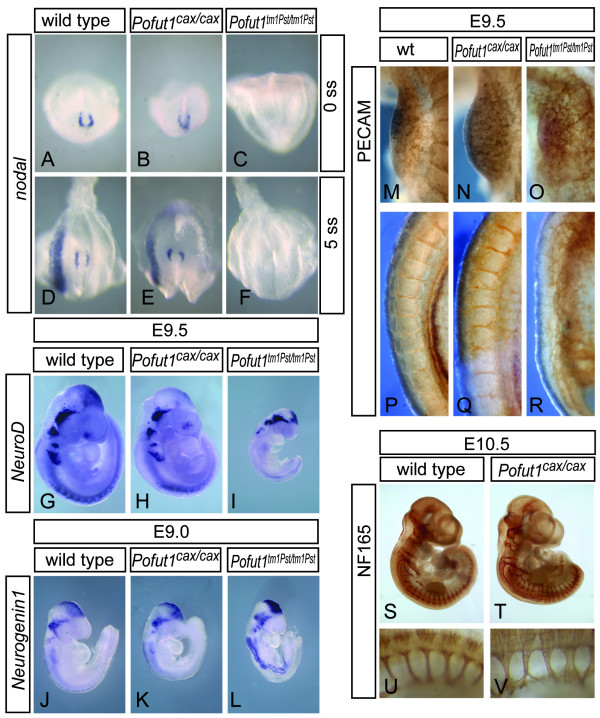
**Apparently normal Notch-dependent early developmental processes**. Expression of Nodal in *Pofut1*^*cax*/*cax *^mutants (B, E) is identical to wild type (A, D) at E8 (A-C) and 8.5 (D-F) indicating undisturbed left-right determination in contrast to *Pofut1*^*tm*1*Pst*/*tm*1*Pst *^embryos (C, F) that lack nodal expression completely. (G-L) Whole mount in situ hybridization of wild type (G, J), *Pofut1*^*cax*/*cax *^(H, K), and *Pofut1tm1Pst*^/*tm*1*Pst *^(I, L) embryos with *NeuroD *at E9.5 (G-I) and *Neurogenin1 *at E9 (J-L) as well as whole mount immunohistochemistry with anti NF160 antibody at E10.5 (S-V) indicates apparently normal neuronal differentiation in *Pofut1 *^*cax*/*cax *^embryos in contrast to *Pofut1tm1Pst*^/*tm*1*Pst *^embryos that show upregulation of NeuroD (I) and Neurogenin1 (L) indicative of enhanced neuronal differentiation. (M-R) Whole mount immunohistochemistry with anti-PECAM antibody at E9.5 showing the limb bud region (M-O) and intersomitic vessels (P-R). In *Pofut1*^*cax*/*cax *^mutant embryos the vascular network was virtually identical to wild type (N) and showed only minor irregularities in intersomitic vessels (Q), whereas vessels in *Pofut1*^*tm*1*Pst*/*tm*1*Pst *^embryos were severely disorganized (O, R).

To address whether also other early processes regulated by Notch are affected by the *cax *mutation we first analyzed establishment of left-right asymmetry, the earliest reported developmental process requiring Notch activity in mice [34,35]. Loss of POFUT1 activity appears to completely block all early Notch signalling [19,36]. However, thus far a requirement for POFUT1 during establishment of left-right asymmetry has not been evaluated. Consistent with an absolute requirement of POFUT1 for Notch activity, *Pofut1*^*tm*1*Pst*/*tm*1*Pst *^embryos (n = 6) showed defects in establishment of left-right asymmetry as indicated by the loss of *Nodal *expression (Figure 6C, F), which is directly regulated by Notch signalling [35]. In contrast, none out of 25 *Pofut1*^*cax*/*cax *^embryos showed abnormal *Nodal *expression (Figure 6B, E), and 50 analyzed heteroallelic *Pofut1*^*Tm*1*Pst*/*cax *^embryos at E9.5 had normal turning and heart looping (data not shown), suggesting that POFUT1 activity derived from one hypomorphic allele modifies Notch signalling to a level sufficient for this process. Vascular remodelling is another early process that critically depends on Notch signalling [37-41] and *Pofut1*^*tm*1*Pst*/*tm*1*Pst *^embryos (n = 6) show severe vascular malformations [19]. PECAM1 staining showed a highly irregular vascular network in *Pofut1 *null mutant embryos in all regions of the body (Figure 6O, R, and data not shown). In contrast, *Pofut1*^*cax*/*cax *^embryos had a vascular network that was virtually identical to wild type embryos (Figure 6N, Q), except for occasional minor irregularities of the intersomitic vessels, which most likely arise secondarily to somite patterning defects. In addition, overall neuronal differentiation assessed by expression of neurofilament (NF165) as a pan-neuronal marker was not obviously affected by reduced *Pofut1 *expression (Figure 6T, V) except for irregular spacing and width of spinal nerves (Figure 6V), which also are most likely secondary to and indicative of disrupted somite patterning. Likewise, expression of *NeuroD *and *Neurogenin1 *was apparently unaltered in *cax *mutants (Figure 6H, K) whereas both genes were clearly upregulated in *Pofut1 *null mutants (Figure 6I, L) indicative of enhanced neuronal differentiation due to loss of Notch activity. Thus, establishment of left-right asymmetry, angiogenesis and neuronal differentiation proceed apparently normal in *cax *mutant embryos whereas *Lfng *expression appeared abnormal in most mutants and anterior-posterior somite patterning was consistently affected.

## Discussion

We have identified a novel allele, *Pofut1*^*cax*^, of the mouse *Pofut1 *gene that leads to reduced *Pofut1 *mRNA and protein levels. Reduction of POFUT1 in embryos homozygous for this allele consistently affects anterior-posterior somite patterning, which at least in part is due to abnormal *Lfng *expression in the anterior PSM, but apparently has no impact on other early developmental processes outside the paraxial mesoderm known to be dependent on Notch signalling. Our data suggest that Notch signalling in distinct developmental contexts is differentially sensitive to the levels of POFUT1 and/or POFUT1-dependent modifications.

The complementation test in conjunction with the map position of *Pofut1*^*cax *^and the intermediate phenotype of *Pofut*^*tm*1*Pst*^*/Pofut1*^*cax *^heteroallelic embryos demonstrated that *cax *is an allele of *Pofut1 *and that the *cax *mutation leads to reduced *Pofut1 *function. Since the coding sequence of *Pofut1 *is not altered in the *Pofut1*^*cax *^allele, the enzymatic properties of POFUT1 are not affected. However, we observed a significant variable reduction of *Pofut1 *mRNA and protein, which provides a plausible explanation for reduced POFUT1 activity. Most likely the IAP insertion that occurred close to the 3' end of intron 4 is responsible for reduced *Pofut1 *mRNA either by interfering with transcription or by destabilizing the message. Insertional mutagenesis by IAPs is not uncommon [42-45], and other insertions into introns that cause mutations have been reported [43,46]. The C3H/He inbred strain of mice appears to have a particularly high frequency of IAP insertional mutations [43,44,46].

Whereas loss of POFUT1-mediated Notch modification appears to block all Notch activity (this paper and [19]), reduced POFUT1 levels in embryos homozygous for the *Pofut1*^*cax *^allele affects predominantly and consistently anterior-posterior somite patterning. Disruption of normal cyclic *Lfng *expression in the PSM likely contributes to these abnormalities since overexpression or interfering with the cyclic expression of *Lfng *was shown to cause somite compartmentalization defects similar to the loss of *Lfng *function [30,33,47]. One potential explanation for the apparent high sensitivity of Notch signalling during somitogenesis to *Pofut1 *levels could be that normal *Pofut1 *mRNA levels are particularly low in the presomitic mesoderm (PSM), where Notch signalling is critical for somite patterning, and *Pofut1 *levels in mutants fall only in the PSM below a critical threshold. However, substantial differences in expression levels in different tissues were not apparent in wild type embryos at E9.5 (Figure 2D a, c), a stage at which *cax *mutants showed already substantial defects in their somites. However, we cannot exclude that such differences may exist but were not detected by the limited quantitative resolution of *in situ *hybridization.

In *Drosophila*, the POFUT1 protein appears to be required for efficient presentation of Notch at the cell surface [23] and/or for the constitutive trafficking of the Notch receptor to early endosomes and downregulation of signalling [48]. It has been proposed that POFUT1 also acts in the mouse PSM as a chaperone that is essential for Notch1 presentation at the cell surface [24], whereas in CHO and ES cells POFUT1 was not required for stable surface expression but for ligand binding and Notch activation [49]. If POFUT1 is required for Notch presentation at the cell surface in the PSM reduced POFUT1 levels might cause reduced Notch levels at the cell surface, which in turn could lead to attenuated Notch activity. If that were indeed the case one would have to assume that Notch trafficking in the PSM is particularly sensitive to POFUT1 protein levels, for which there is no experimental evidence at present.

Alternatively, different fucosylation sites may require different levels of POFUT1 activity for efficient modification, and reduced POFUT1 levels might affect some sites more than others. Since conserved O-fucosylation sites may have distinct functions with respect to Notch activation and/or trafficking [50], differential O-fucosylation may result in context-dependent effects. Such effects have indeed been observed for the Notch1 receptor in mice, where mutation of the O-fucosylation site in EGF repeat 12, which is essential for ligand binding, results in a hypomorphic allele which is compatible with apparently normal embryonic development but affects post-natal growth and T-cell development [51]. Context dependent effects might also depend on the role of O-linked fucose residues as substrates for further modifications by FNG glycosyltransferases. In mice, there are three fringe proteins, LFNG, RFNG and MFNG, which are expressed in distinct patterns during development [52-54]. Loss of RFNG has no obvious consequences [55], no in vivo data on the function of MFNG have been reported, and loss of LFNG leads to severe anterior-posterior somite patterning defects [30,56] suggesting that FNG modification of Notch is of particular importance for somite patterning. Since fringe proteins modify different regions of Notch in vitro [57], and not all O-fucosylated EGF repeats are substrates for fringe activity [21], reduced O-fucosylation might preferentially affect sites that are substrates for LFNG and thus important for somitogenesis.

Our findings and conclusions conflict with previous findings suggesting that somite segmentation is less sensitive to reduced Notch activity than neural tissue [58]. In these experiments, a Notch allele was used that gives rise to a processing-defective Notch protein. This mutant Notch protein can be processed to NICD by an unidentified protease more effectively than wild type Notch, and it was suggested that this protease is more active in the paraxial mesoderm [58]. Thus, processing-defective Notch could generate more residual Notch activity in the PSM than in neural tissue providing the basis for the mild somite defects observed by Huppert at *al*. [58]. In addition, segment border formation was used as the major criterion, but anterior-posterior patterning was in general more affected than segmentation (border formation) in their studies. Since Notch activity is not essential for border formation, but pivotal for anterior-posterior somite patterning [32], the results of Huppert et *al. *[58] could also be interpreted in favour of a high sensitivity of somite compartmentalization to reduced Notch activity and POFUT1 levels that we observed in *cax *mutant embryos.

## Conclusion

Reduction of *Pofut1 *expression to approximately 25% affects expression of Notch target genes and Notch-dependent processes differently in different tissues. Cyclic *Lfng *expression and anterior-posterior somite patterning is highly sensitive to the level of POFUT1 in early mammalian embryos whereas other early Notch-dependent processes apparently are not. Reduced POFUT1 levels might affect trafficking and/or O-fucosylation of Notch as well as its further modification by LFNG due to abnormal *Lfng *expression. Since FNG modification of Notch appears to be of particular importance for somite patterning, and not all O-fucosylated EGF repeats are substrates for Fringe activity, we propose that reduced O-fucosylation might preferentially affect sites that are substrates for LFNG and thus important for somitogenesis. The hypomorphic *Pofut1*^*cax *^allele should facilitate to further dissect the roles of POFUT1 for Notch signalling in different developmental contexts and at later stages of development.

## Methods

### Mice

The recessive *cax *mutation arose spontaneously in a C3H/HeJ colony of mice at The Jackson Laboratory and has been maintained on this strain background. The inbred mouse strain carrying the mutation is designated C3H/HeJ-*Pofut1*^*cax*^/J, Jackson Laboratory Stock Number 7782. Phenotypic analysis was performed with *cax *mutants on a mixed genetic background due to the low breeding performance of the inbred line.

### Genetic mapping

Intersubspecific F1 hybrids were generated by mating C3H/HeJ-*cax/cax *mutants with CAST/Ei-+/+ mice. An intercross of these F1 hybrids produced 62 F2 mice with mutant phenotypes (*cax/cax *genotype) that were used to determine the initial map position of the *cax *mutation. A backcross of (C3H/BL6xCAST/Ei) F1 hybrids to C3H/B6-*cax/cax *mutant mice produced 1339 N2 mice with an unambiguous mutant tail phenotype that were analyzed for high resolution mapping. Initially, we mapped *cax *with respect to the flanking simple sequence length polymorphism (SSLP) markers *D2Mit22 *and *D2Mit409 *using all 1339 backcross animals. We identified 62 recombinants, which were genotyped for SSLP markers located between *D2Mit22 *and *D2Mit409*.

### In situ hybridization

Whole mount in situ hybridization was performed according to [59]. The probes used were originally obtained from Dr. Manfred Gessler (*Hey1, HeyL*), Dr. Martyn Goulding (*NeuroD, neurogenin*), Dr. Tom Gridley (*Lfng*), Dr. Ryoichiro Kageyama (*Hes1, Hes7*), Dr. Andreas Kispert (*Uncx4.1, Tbx18*), Dr. Janet Rossant (*Cer1, nodal*), Dr. Yumiko Saga (*Mesp2*), or isolated in our laboratory (*Dll1, Pofut1, Papc*).

### Histology

Embryos were fixed in 4% PFA, dehydrated, embedded in paraffin, sectioned at 10 μm, stained with Nuclear Fast Red, and embedded in Mowiol (Calbiochem).

### Immunohistochemistry

For immunohistochemical analysis embryos were fixed in 4% paraformaldehyde, and in case of NF165 staining subsequently treated with proteinase K. Primary antibodies used were anti-PECAM (Pharmingen) diluted 1:100, and anti-NF165 (DSHB clone 2H3) diluted 1:50. Secondary antibodies were biotinylated anti-rat and anti-mouse (Vectastain) diluted 1:200. The signal was intensified with the ABC system (Vectastain) and bound antibodies were detected with DAB (Sigma).

### PCR Analysis of *Pofut1 *mRNA

To search for mutations in the open reading frame of Pofut1 mRNA from C3H/HeJ-*cax/cax *or C3H/HeJ embryos was prepared either with magnetic Dynabeads (Dynal Novagen) or with a Direct mRNA kit (Qiagen). cDNA was synthesised with Superscript II Reverse transcriptase (Invitrogen or Promega). cDNA was amplified in two overlapping fragments using the following gene specific primers: for Exon 2-Exon4: CTG CTT CTG CTG CTG TTG CTG C and CAG TGC GAG CAC AGG ATG CTC, and for Exon 5-Exon7: CAA TGG ACC CAG AGA TTT CCT GCA and GGT TGA GGG TGG GAG GTG GG. Exon1 was amplified from genomic DNA with the primers GCC ATT GTG CGG TGC ATT G and AAG CAG AGG GTT CCG GAG GC. PCR fragments from at least two independent PCR reactions were subcloned either into TpGEM easy or pCRII Topo vectors and sequenced.

### PCR Analysis of genomic DNA

To identify gross abnormalities of the Pofut1 gene about 20 kb of the promoter region, the introns and the untranslated 3'region were amplified by PCR as overlapping fragments of about 0.5 to 1 kb in length. Primer sequences were selected based upon . Primer sequences used to amplify the fragments shown in the figures are as follows: fragment 4/10: TCCATTTTGCCCTTTCAAAGGT and ACACAGAATCCTTTCTGCAATCTTTC; fragment 4/11: GCACTGCCACTGGGGCTAGT and CCCAGGCAGTGCGAGCA; fragment E5: AGATTTCCTGCAAAAGAGCATCCT and GAGCTAAAATCCAGACTTGGTGGA; fragment 5/1: CATTCATCTGCGCATTGGCT and AGTGGGACTGCAGATCACTCCC. Sequences of all other primers are available upon request.

A DNA fragment containing the insertion in the *cax *mutation was amplified with the long range PCR Kit from Qiagen using primers GCACTGCCACTGGGGCTAGT and CCCAGGCAGTGCGAGCA, followed by a nested PCR reaction with primers GAAAGATTGCAGAAAGGATTCTGT and AGGATGCTCTTTTGCAGGAAATCT. The fragment was subcloned in the TopoXL vector (Invitrogen) and sequenced. Primer pairs used to specifically detect the 5' and 3' ends of the IAP insertion in the *cax *allele were: AGGGCTCTTTTTGCGTCCTGT and TGGCGCTGACATCCTGTGTT, and CCCAGGCAGTGCGAGCA and TCAAGATCAGACTTACCTCGTTCC, respectively.

### Southern Blot hybridization

Southern Blot hybridizations were performed according to standard procedures with a *Pofut1 *cDNA probe containing exons 5 and 6 and the 5' region of exon7.

### Quantitative Real-time PCR analysis

RNA was prepared from individual embryos using the RNeasy Minikit (Quiagen), and reverse transcribed using the High Capacity cDNA Reverse Transcription Kit (Applied Biosystems) according to the manufacturers' instructions. *Pofut1 *cDNA was quantified on an ABI 7900HT using two gene specific assays detecting portions of exons 1 and 2, and exons 6 and 7, respectively, (TaqMan gene expression assays Mm 01240157 m1 (for *Pofut1 *exon 6–7), Mm 00475567 m1 (for *Pofut1 *exon 1–2)) each measured in quadruplicate using wild type embryo cDNA as biological calibrator, GAPDH and HPRT as endogenous controls (TaqMan gene expression assays Mm 00446968 m1 (for *Hprt *exon 6–7), Mm 999999159 g1 (*Gapdh *exon 1–2), and additional no-template, no reverse transcription and blank controls. Data were evaluated using the SDS RQ Manager (V1.2 ABI) and the delta-delta Ct method [60,61]. Analysis of variance (ANOVA) was carried out using the statistical software package BIAS for Windows Version 8.6.3.

### Western Blot analyses

12 wild type and cax mutant embryos, respectively, and four *Pofut1*^*tm*1*Pst*/*tm*1*PSt *^E9.5 embryos were pooled, and lysed in 2× Lämmli buffer. The equivalent of one embryo from pooled embryo lysates was loaded per lane. Proteins were separated by SDS-polyacrylamid gel electrophoresis and blotted onto transfer membranes (Immobilon, Millipore). After blotting membranes were incubated with Qentix™ Western Blot Signal Enhancer (Pierce), blocked with 10% milk powder in TBST at 4°C over night, incubated with rabbit anti-POFUT1 antibodies [29] diluted 1:1000 in TBST with 1% milk powder for 3 hr at 4°C, washed five times in TBST, incubated with HRPOD conjugated donkey anti-rabbit (GE Healthcare, 1:10000 in TBST with 1% milk powder), and washed five times in TBST. Bound antibodies were detected using ECL Western Blotting Detection Reagents (GE Healthcare) using a Fujifilm LAS 3000 gathering signals every 3 min over an 21 minute interval.

## Authors' contributions

KSG did the backcross analysis and fine genetic mapping, planned and performed the molecular characterization of the *cax *allele, did the phenotypic analyses, TaqMan PCR and Western blot analysis, and worked on the manuscript. JS helped with the TaqMan measurements and did the statistical analyses. BH identified the mutant, analyzed inheritance and worked together with KRJ on the chromosomal assignment and corrections of the manuscript. AG conceived the study, planned experiments, and drafted the manuscript. All authors read and approved the final manuscript.
